# Correction: FeS colloids – formation and mobilization pathways in natural waters

**DOI:** 10.1039/d4en90048k

**Published:** 2024-11-05

**Authors:** Vincent Noël, Naresh Kumar, Kristin Boye, Lilia Barragan, Juan S. Lezama-Pacheco, Rosalie Chu, Nikola Tolic, Gordon E. Brown, John R. Bargar

**Affiliations:** a Stanford Synchrotron Radiation Lightsource, SLAC National Accelerator Laboratory 2575 Sand Hill Road Menlo Park CA 94025 USA noel@slac.stanford.edu; b Department of Geological Sciences, School of Earth, Energy & Environmental Sciences, Stanford University Stanford CA 94305-2115 USA; c Department of Environmental Geosciences, Centre for Microbiology and Environmental Systems Science, University of Vienna 1090 Vienna Austria; d Center for Environmental Implications of NanoTechnology (CEINT), Duke University P.O. Box 90287 Durham NC 27708-0287 USA; e Department of Earth System Science, Stanford University Stanford CA 94305 USA; f Environmental Molecular Sciences Laboratory, Pacific Northwest National Laboratory Richland WA 99354 USA

## Abstract

Correction for ‘FeS colloids – formation and mobilization pathways in natural waters’ by Vincent Noël *et al.*, *Environ. Sci.: Nano*, 2020, **7**, 2102–2116, https://doi.org/10.1039/C9EN01427F.

The authors regret that they did not make the connection between this work and the work in ref. 41 previously published by the same group of authors clear.

In the Introduction, the section beginning with “The recent study by Kumar…” should have read:

In our previous study^41^ we found that the ratio of dissolved sulfide to iron (sulfide/Fe) is a critical variable in the Fh sulfidation reaction, where we only focused on the solid phase transformation. Reductive dissolution of Fh resulted in incremental releases of dissolved Fe(ii) up to a sulfide/Fe ratio of 0.5, with Fe(ii) concentrations declining sharply above this ratio, suggesting formation and settling of poorly crystalline iron monosulfide (FeS). However, dissolved Fe(ii) is not thermodynamically stable in waters at circumneutral pH values and should react with aqueous sulfide, resulting in the formation of aqueous FeS complexes as well as larger colloidal particles.^42^ Aqueous FeS clusters, defined operationally in terms of their voltammetric characteristics, have been previously detected in natural (sub)surface water.^33,42,43^ Thus, we posit that aqueous FeS clusters and/or larger colloidal particles are able to resist aggregation that results in precipitation and can potentially sorb nutrients and contaminants and facilitate their transport under conditions outside of the solubility regime of the individual participating ions. Building upon our previous work which focused only on solid phase transformation,^41^ in this study, we used selected S/Fe ratios to focus on the characterization of FeS colloids in aqueous phase. We found that the Fh sulfidation reaction indeed generates FeS clusters and larger colloidal particles and determined the conditions required for their stability in aqueous suspensions.

In the section 3.5 TEM characterization of solid fraction, this additional sentence should have been included:

The change in solid solution ratio (200 mg ferrihydrite in 20 mL against 200 mg ferrihydrite in 65 mL) is primarily due to the analysis volume needed in the [present] experiment. Based on the well-supported assumption that the reaction is governed only by S/Fe ratios, we compare results and reinterpret the previous TEM image.

In the 2.1.1 Ferrihydrite synthesis section, this should have read as:

Ferrihydrite was synthesized following the method described in Kumar *et al.*^41^

The Fig. 7 caption should have read:

TEM images of the solid fraction (Fig. 1) from sulfidation of Fh in a 0.1 M NaCl solution after 336 h for sulfide ratio of 0.5 (a) and 2 (b) reinterpreted from Kumar *et al.*^41^ The white circles and lines show the nanoparticles and the orientation of layers, respectively.

In the Discussion section 4.1, the sentences beginning “These results suggest that aqueous…” should have read:

These results suggest that aqueous FeS clusters serve as precursors of larger FeS colloidal particles, which necessitates the following modification to our model proposed earlier^41^ for Fh sulfidation at low sulfide/Fe ratios. Fe(ii) released from the reductive dissolution of Fh by dissolved sulfide produces aqueous FeS clusters at low sulfide/Fe ratios.

The Royal Society of Chemistry apologises for these errors and any consequent inconvenience to authors and readers.

